# Has the opening of Amazon fulfillment centers affected demand for disability insurance?

**DOI:** 10.1371/journal.pone.0294453

**Published:** 2023-11-27

**Authors:** Kara E. Rudolph, Nicholas T. Williams, Floriana Milazzo, Atheendar Venkataramani, Rourke O’Brien

**Affiliations:** 1 Department of Epidemiology, Mailman School of Public Health, Columbia University, New York, New York, United States of America; 2 Departments of Health Policy and Medicine, University of Pennsylvania, Philadelphia, Pennsylvania, United States of America; 3 Department of Sociology, Yale University, New Haven, Connecticut, United States of America; Sungkyunkwan University School of Social Sciences, KOREA, REPUBLIC OF

## Abstract

An estimated 17.6% of blue-collar, manufacturing jobs were lost in the United States between 1970 and 2016. These jobs, often union-represented, provided relatively generous pay and benefits, creating a path to the middle class for individuals without a four-year college degree. Evidence suggests the closure of manufacturing facilities and resulting decline in economic opportunity increased demand for disability insurance (SSDI) among blue-collar workers. In recent years, the opening of Amazon Fulfillment Centers (FCs) has accelerated around the country, driving a wave of blue-collar job creation. We estimated the extent to which the opening of FCs affected SSDI application rates, including rates of approvals and denials, using a synthetic control group approach. We found that FC openings were associated with a 1.4% reduction in the SSDI application rate over the subsequent three years, translating to 5,528 fewer applications per year across commuting zones with an FC opening. Our findings are consistent with FC openings improving economic opportunities in local labor markets, though our confidence intervals were wide and included the null.

## Introduction

An estimated 17.6% of blue-collar, manufacturing jobs were lost in the United States (US) in the period 1970–2016 [1]. These jobs, which were largely union-represented [2], created a path to the middle class for non-college-educated individuals by providing relatively generous pay, including overtime pay, and benefits [3, 4]. The decline of manufacturing created a void in local labor markets, depressing demand for workers without a bachelors degree or higher.

Rising unemployment rates and decreasing labor force participation rates have accompanied these job losses [5], concentrated in areas experiencing deindustrialization and an erosion of economic opportunity for non-college-educated workers [6]. (Of note, unemployment and labor force participation are distinct: someone who is unemployed and has actively looked for work during the previous four weeks is counted as “unemployed” [7], whereas someone who is unemployed and has not actively looked for work during that time period is counted as having dropped out of the labor force.) Those who desire work but have dropped out of the labor force have been termed “discouraged workers” [8]. A lack of economic opportunity can push discouraged workers into early retirement [9], back to school [9, 10], or onto disability insurance [9, 11]. Disabled workers in the US can apply for Social Security Disability Insurance (SSDI) without an income or asset requirement or Supplemental Security Income (SSI) if they are low-income with limited assets. Both SSDI and SSI provide income support; SSI also provides health insurance through Medicaid [12].

As with labor force participation, SSDI applications are correlated with labor market cycles, including unemployment rates and recessions [13–16]. SSDI applications may be especially correlated with the blue-collar job loss due to the increased prevalence of SSDI receipt with decreasing levels of education [17]. Indeed, SSDI applications have been found to be related to the loss of manufacturing jobs [18], increases in automation and robotization in the manufacturing sector [18], and even the boom-bust cycles of the coal mining industry [19].

However, the association between SSDI applications and economic opportunity may be surprising, because SSDI is designed to provide income in cases of a significant disability incompatible with work, which would seem unrelated to economic opportunity. There could be several, non-mutually-exclusive hypothesized explanations for this relationship.

The first such hypothesized explanation is that there exists a subgroup of workers with health impairments or a disability, called “conditional applicants”, who would apply for SSDI if they lost their job [13, 15]. These workers may be able to do some jobs with their disability, including their current job, but such jobs may not be available in a constrained economic environment. There is support for this explanation in the literature, including that applicants who apply during economic downturns are more likely to be higher-earners with more recent work experience [16, 20]. In addition, several research groups have found that increases in SSDI applications in response to worsening economic conditions are primarily comprised of applications that are initially denied [13, 18].

A second hypothesized explanation is that the prevalence of true disability—the inability to meet self-care needs or carry out routine tasks—is increasing among working-age adults on a macro level [17, 21], concurrently with deindustrialization, and on a more local level in economically constrained environments (using measures of self-reported disability) [22, 23]. For example, during a period of deteriorating economic opportunity, the jobs that are available may be less safe with fewer worker protections in terms of occupational safety, resulting in more injuries. There is also some support for this explanation in the literature. Americans in mid-life who are unable to meet self-care needs or carry out routine tasks has increased markedly in recent decades, especially for those with a high school degree or less [24–26]. In addition, accompanying the decline in blue-collar jobs has been a concomitant decline in unionization and the protections it afforded [27]. For example, in Michigan, 35% of workers were union members in 1980, but only 14.7% in 2014.

The opening of Amazon fulfillment centers (FCs) may impact demand for SSDI through both of the above explanations. FCs are large (typically 800,000 square feet) facilities where more than 1,000 workers (typically 1000–2500, 1500 on average) sort, pack, and ship Amazon orders [28, 29]. The opening of FCs has accelerated in recent years, providing a rare example of blue-collar job creation. Economists have found that the opening of FCs increase the number of jobs in the warehousing industry [29, 30], including by more than expected from the FC alone, suggesting spillover effects [29, 30]. However, the effects on overall county-level employment have only been detected in the fourth quarter (if at all [30]), reflecting seasonal job creation [29].

There are several parallels between FCs and manufacturing plants. First, both employ large numbers of workers who do not need a college degree and/or without any specialized training. Second, both employ workers to perform largely manual labor. Workers at FCs report working 10–12 hour days, walking between 7–15 miles a day, and lifting up to 50 pounds [31]. However, there are also marked differences. For example, until 2022, no FC had unionized workers. Workers at a Staten Island FC unionized in April 2022; this remains the only FC with unionized workers. In contrast, an estimated 48.4% of workers at auto manufacturing plants were unionized in 1990 and 71% were unionized in 1973 [32].

In this paper, we estimated the extent to which the opening of FCs affected SSDI application rates. We hypothesized that application rates would decrease immediately following an opening but that this initial decline would attenuate over time due to increasing workplace injuries. We also hypothesized that the initial decline in applications would be driven by a decline in applications that were denied and that the attenuation of that decline over time would be driven by an increase in approved applications. Consequently, we also estimated the extent to which FC openings affected the rate of denied SSDI applications and the rate of approved SSDI applications. However, we treated these analyses as exploratory, because application decisions can be made years after the submission, and many initially denied applications (estimates of approximately 50% [33]) may ultimately be awarded, meaning that our data on SSDI applications and determinations would be incomplete for the more recent years of data. To disentangle and isolate changes and trends in SSDI application rates, including denials and approvals, that are attributable to FC openings in particular, we used a newly developed partially pooled synthetic control group approach [34] to estimate the effect of the staggered opening of Amazon FCs from 2006 to 2017 on rates of SSDI applications over the subsequent 3 years.

## Materials and methods

### Sample

We used data 2001–2017 from *N* = 380 commuting zones (CZs, out of 709 CZs nationally) that were in metro areas of any size or nonmetro areas that were adjacent to a metro area with an urban population greater that 20,000 (corresponding to rural-urban continuum codes 1–4), because all FC openings were in such areas. We used commuting zone as the level of analysis, as this represents the local labor market. However, we use counties as the unit in a sensitivity analysis. CZs in Alaska were excluded because of boundary changes during the time period. Within a commuting zone, we excluded counties with missing outcome data (3.4% of counties) and with missing economic mobility data (an additional 0.5% of counties). This secondary analysis of deidentified data was deemed to be nonhuman subjects research by the Columbia University Institutional Review Board, and consequently, informed consent was not applicable.

### Measures

Measures consisted of covariates, FC openings as the exposure, and outcomes of SSDI applications, approvals, and denials.

#### Covariates

Covariates included: median household earnings, proportion male; proportion white, black, Hispanic/Latino; distribution of educational attainment; population density; age distribution; proportion unemployed; proportion not in the labor force; proportion employed in manufacturing jobs; distribution of marital status; and proportion veterans, all obtained from the US Census Bureau. Lastly, we also used an index of economic mobility as a covariate [35]. We provide additional detail in Section S1 of the S1 File.

#### Exposure

We obtained a list of Amazon Fulfillment Centers from https://sellercentral.amazon.com/gp/help/external/201811680?language=en-US&ref=mpbc_200240440_cont_201811680. Data on FC openings was obtained from https://mwpvl.com/html/amazon_com.html. We included openings that occurred between 2006–2017. For CZs that experienced multiple FC openings in this time period, we use the date of the first opening. This resulted in between 5 and 16 years of data before the FC opening, with an average of 12.47 years.

#### Outcome

The number of SSDI applications, denials, and approvals for adults aged 18–64 years by county for years 2001–2017 were obtained from the Social Security Administration and aggregated to the commuting zone level and rates per 1,000 residents 12 years or older were calculated. The outcomes were log-transformed prior to the analysis. In a sensitivity analysis, we also used the number of disabled workers currently receiving SSDI by commuting zone as an outcome, obtained from https://www.ssa.gov/policy/docs/statcomps/oasdi_sc/2000/index.html.

### Statistical analysis

There are several analytic options in the current setting where we have panel data and multiple units experiencing the treatment (in this case, opening an FC) over time, the most common of which include 1) two-way fixed effects (TWFE) linear regression, 2) staggered adoption difference-in-differences (DiD), 3) sequential DiD, and 4) staggered adoption synthetic control method (SCM). 1) TWFE regression assumes: a) independent and identically distributed units without interference; b) that the linear regression outcome model is correctly parametrically specified, meaning that that the two types of unobserved confounders (unit-specific and time-specific) contribute in a linear, additive way [36]; c) no anticipation, meaning that the intervention has no effect on the outcomes before implementation; and d) that there is no time-varying confounding. Assumptions c and d together are the “parallel trends” assumption [37]. A criticism of TWFE is that it may not consistently estimate the average treatment effect on the treated (ATT) in the presence of treatment effect heterogeneity [36, 38–40]. 2) Staggered adoption DiD typically assumes the same set of assumptions as TWFE regression, {a, b, c, d} (though may rely on less strict parametric assumptions [41]), but can estimate each cohort-specific (group of units receiving the intervention at the same time) ATT consistently [40, 41]. 3) Sequential DiD relaxes assumption d from above and allows for time-varying confounders that change at the same rate between treatment and control groups (together with the “no anticipation” assumption, it assumes parallel trends-in-trends) [42]. Lastly, 4) staggered adoption SCM assumes {a, c} from above, one of two assumptions about the outcome process: either that the outcomes are generated under a e) linear latent factor model that assumes no unobserved confounding related to treatment timing or f) auto-regressive model that assumes no unobserved confounding in the post-treatment period that is related to treatment timing, and g) a well-fitting synthetic control for either every unit (in the case of a unit-specific SCM) or for the average across units (in the case of the pooled SCM) can be found based on a weighted control fit of the outcome model in the pre-intervention period [34, 43]. We note accurately fitting pre-intervention trends requires an adequately long pre-intervention period [43].

We wished to make the fewest and least restrictive assumptions, so chose the staggered adoption synthetic control approach. At each year of an FC opening, the staggered-adoption SCM created a weighted combination of CZs that did not have an existing FC—the synthetic control—such that their pre-FC outcomes and pre-FC averaged covariate values match those of the CZ(s) with an FC opening that year as closely as possible. As recommended, we give equal weight to the covariate and outcome imbalance [34].

Specifically, we used a partially pooled staggered adoption SCM that makes several extensions to the approach described above [34]. First, it uses unit-level fixed effects, proposed previously [44], essentially de-meaning the outcome trends (i.e., relaxing the zero-intercept restriction of the original synthetic control [43]). Second, it combines two SCM approaches: 1) “separate SCM”, which estimates weights that optimize the match in outcome and covariates between the treated unit and weighted controls (the synthetic control) in the pre-intervention period for each treated unit separately; and 2) “pooled SCM”, which estimates weights that optimize the match in the outcome and covariates between all the treated units averaged together and weighted controls. It combines the two by minimizing a weighted average of the separate SCM and pooled SCM imbalances in pre-FC fit, where the pooling parameter determines the relative weight given to each imbalance. As recommended, we chose the value of the pooling parameter to be the ratio of the pooled fit over the separate SCM fit [34], though assess the sensitivity of our results to alternative parameter values. The partially pooled implementation relaxes the unit-specific approach’s assumption that a well-fitting synthetic control can be found for every unit and relaxes the pooled approach’s assumption that the data generating process does not change over time [34]. Third, it uses the wild-bootstrap to obtain pointwise 95% confidence intervals [34, 45].

We used the augsynth package in R. Code to replicate our analysis is available: https://github.com/kararudolph/code-for-papers/blob/master/amazon.R.

## Results

Table 1 gives the year of each FC opening and closing from 2006 to 2017. Table S1 in the S1 File gives the number of counties with an FC opening by state.

**Table 1:** Number of FC openings and closings between 2006 and 2017.

Year	New FCs	Closed FCs
2006	2	0
2007	1	0
2008	3	0
2009	0	3
2010	4	0
2011	3	1
2012	6	0
2013	6	0
2014	5	0
2015	3	2
2016	14	0
2017	8	0

Table 2 gives descriptive statistics in terms of each of the covariates from the 2000 Census of CZs with FC openings versus those without. We see that CZs with an FC opening have greater population density, higher median household earnings on average, and slightly more residents who identify as Hispanic and slightly fewer who identify as White.

**Table 2:** Commuting zone level descriptive statistics.

Commuting zones	All commuting zones 380	Has fulfillment center 40	No fulfillment center 340
Population over 12	158,175.35 (361,038.58)	580,714.00 (618,243.71)	139,478.95 (333,536.37)
Male (%)	49.15 (1.23)	49.13 (0.79)	49.15 (1.27)
White (%)	81.41 (13.04)	76.83 (9.80)	81.95 (13.28)
Black (%)	10.83 (12.03)	11.72 (8.00)	10.72 (12.43)
Hispanic (%)	7.53 (12.07)	12.17 (11.92)	6.99 (11.99)
Age 5–17 (%)	18.67 (1.87)	18.88 (1.42)	18.65 (1.92)
Age 18–24 (%)	10.87 (4.11)	9.34 (1.19)	11.05 (4.29)
Age 25–34 (%)	13.32 (1.47)	14.51 (1.45)	13.18 (1.41)
Age 35–49 (%)	22.54 (1.75)	23.56 (1.09)	22.42 (1.78)
Age 50–64 (%)	15.06 (1.72)	14.85 (1.19)	15.08 (1.77)
Age >64 (%)	12.93 (2.94)	11.93 (2.82)	13.05 (2.94)
Less than HS edu. (%)	14.23 (5.68)	15.40 (4.81)	14.09 (5.77)
HS edu. (%)	21.04 (3.88)	20.05 (3.35)	21.16 (3.93)
At least college edu. (%)	13.78 (4.25)	16.28 (3.86)	13.49 (4.20)
Not in labor force (%)	28.43 (4.33)	26.65 (2.95)	28.64 (4.42)
Unemployed (%)	5.84 (1.79)	5.40 (1.52)	5.89 (1.82)
Employed in manufacturing (%)	7.01 (3.47)	6.66 (2.81)	7.06 (3.54)
Never married (%)	20.26 (4.02)	20.49 (2.49)	20.23 (4.16)
Married (%)	45.49 (3.02)	44.79 (2.25)	45.57 (3.09)
Civilian adults (%)	10.33 (2.03)	9.84 (1.77)	10.39 (2.05)
Median household earnings	$21294.99 ($3496.01)	$25041.39 ($2776.31)	$20854.23 ($3305.53)
Population density	480.28 (1552.49)	1225.04 (1168.41)	392.66 (1569.85)
Absolute upward mobility	41.63 (3.82)	40.97 (2.50)	41.71 (3.94)

**Fig 1:**
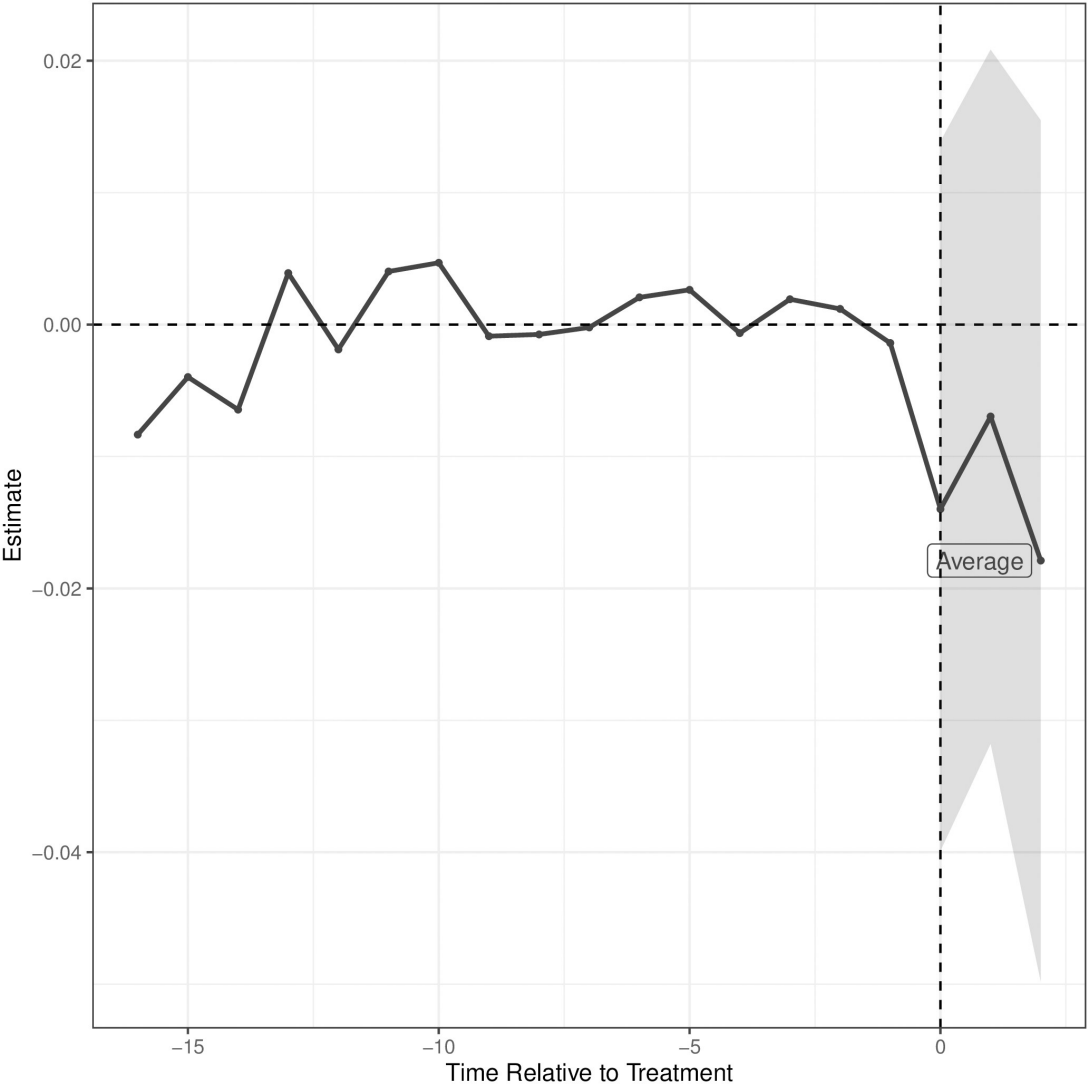
Estimated difference between log-transformed SSDI application rates comparing commuting zones with an FC opening to their synthetic control commuting zones without an FC by year. The synthetic control was a weighted combination of commuting zones without an FC such that the weights minimized the difference in outcome and covariates between FC and non-FC commuting zones in the years preceding an FC opening. The average line to the right of the vertical line represents the effect of an FC opening for the 3 years after the opening, with 95% confidence intervals.

The Y-axis of Fig 1 shows the difference in the log-SSDI application rates between CZs with an FC opening and their synthetic control (the weighted combination of CZs without an FC). The portion of the graph to the left of the vertical line represents years leading up to the opening; the portion of the graph to the right of the vertical line represents years after the opening. We estimate that the opening of a FC results in a 1.4% reduction (95% CI: -4.1%, 1.4%) in the SSDI application rate in the subsequent three years, although this decrease is not statistically significant using the bootstrapped confidence intervals (the shaded portion of the graph). In contrast to expectation, this reduction appears to increase, not attenuate, over subsequent years.

**Fig 2:**
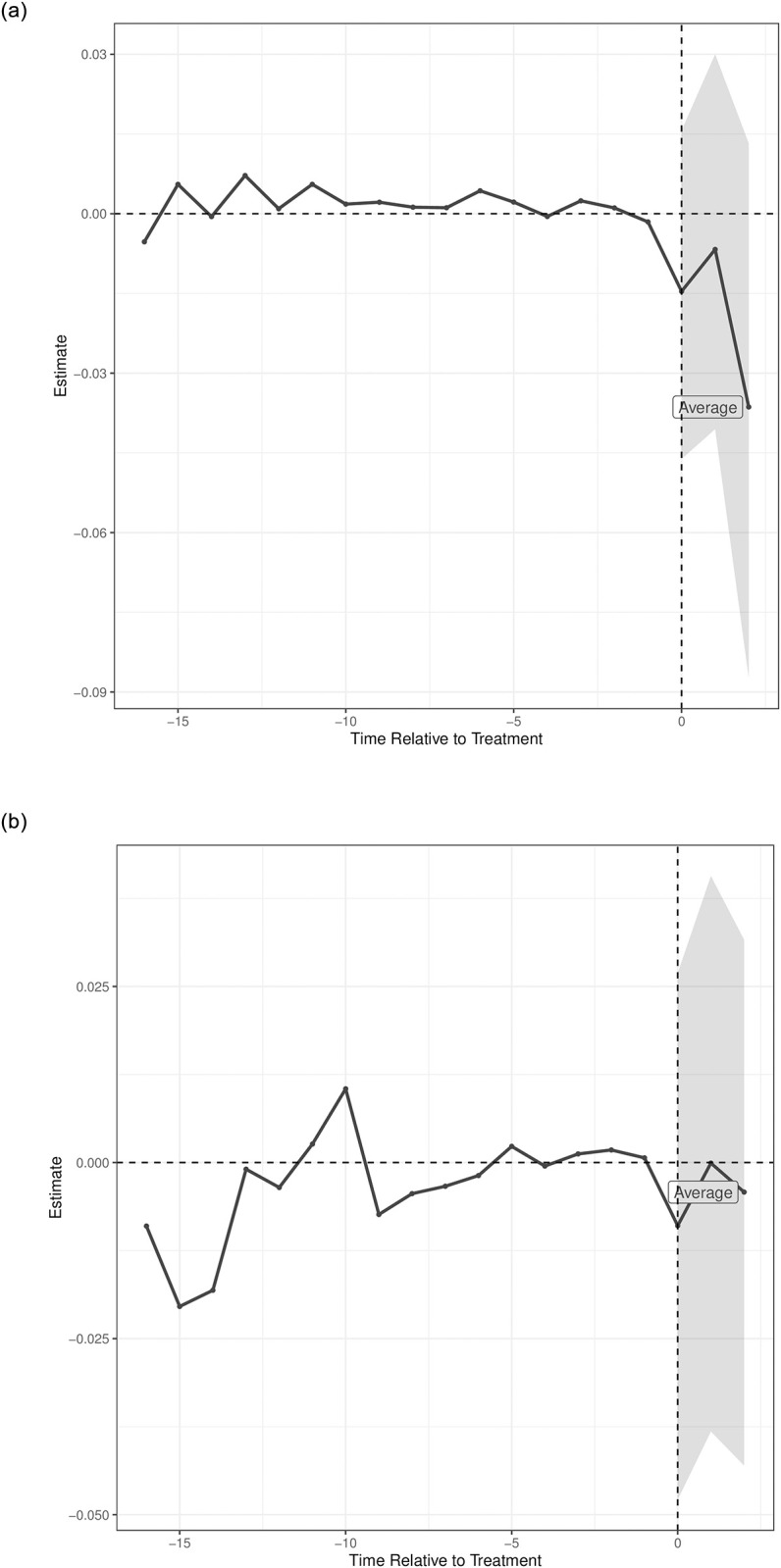
Estimated difference between log-transformed SSDI (a) denial rates and (b) approval rates comparing commuting zones with an FC opening to their synthetic control commuting zones without an FC by year. The synthetic control was a weighted combination of commuting zones without an FC such that the weights minimized the difference in outcome and covariates between FC and non-FC commuting zones in the years preceding an FC opening. The average line to the right of the vertical line represents the effect of an FC opening for the 3 years after the opening, with 95% confidence intervals. (a) SSDI denial rates, (b) SSDI approval rates.

Fig 2 shows the results from the exploratory analysis using SSDI denials (Fig 2a) and approvals (Fig 2b) as the outcomes. FC openings are associated with declining rates of denials, which appears to grow—not attenuate—in subsequent years, though it is not statistically significant. In contrast, FC openings do not appear associated with approval rates.

It is plausible that there is heterogeneity in effects over time that is obscured by our estimates of the ATT. Consequently, Table S2 in S1 File shows the effect estimates by each year cohort of FC openings. Confidence intervals are wide for these cohort-specific effect estimates, but, looking at the point estimates, there is not evidence of appreciable effect heterogeneity with the possible exception of estimates prior to 2011. Previous research has noted that Amazon’s location strategy for FCs changed around 2011 [29], so examining effects after this point is reasonable to reduce heterogeneity. Consequently, we repeated the primary analysis estimating the ATT of FC openings after 2011, and show the results in Fig S1 in S1 File. Results under this sensitivity analysis are similar to those in our primary analysis.

We conducted several additional sensitivity analyses. We assessed the sensitivity to different values of the pooling parameter that determines the relative weights given to the separate SCM and pooled SCM (Fig S2 in S1 File), and saw nearly identical results as in the primary analysis. We also conducted a placebo test by setting the FC openings to be 5 years earlier than actual (Fig S3 in S1 File). As expected, we saw no effect of these fake FC openings. We also conducted our analyses at the county level and saw similar results as in the primary analysis (Fig S4 in S1 File). We conducted our analysis using the number receiving SSDI benefits and also saw similar results as the primary analysis (Fig S5 in S1 File). Because CZs with FC openings may be meaningfully different from CZs never having an FC in ways we have not accounted for using covariates, we repeated the analysis restricting only to those CZs that ever had an FC opening, meaning that CZs with a later FC opening served as controls for those with earlier opening FCs. Results are shown in Fig S6 in S1 File and are similar to the primary analysis.

Lastly, we repeated our analysis using the time-cohort DiD estimator proposed by Callaway et al., 2021 [41]. This estimator makes stronger assumptions than the staggered adoption SCM estimator we use for the primary analysis (see our discussion in the Statistical Analysis section), and there is p-value evidence against the parallel trends assumption in the pre-treatment periods. However, we use this estimator to present an alternative to our SCM analysis. Using this doubly robust DiD estimator on the same data and looking over the same post-treatment period, we estimate the ATT from FC openings on SSDI application rates to be -0.014 (95% CI: -0.029, 0.002), on SSDI denials rates to be -0.020 (95% CI: -0.042, 0.002), and on SSDI allowance rates to be -0.011 (95% CI: -0.037, 0.015). Estimated effects by year are shown in Fig S7 in S1 File, and appear similar to Figs 1 and 2. We also repeat the above sensitivity analysis not restricting the post-treatment period (i.e., allowing estimates up to 11 years post treatment), and show the results in Fig S8 in S1 File. In these figures, we see evidence that effects of FC openings on declines in application rates and denials rates become more pronounced in later years (e.g., years 8–11 post-FC opening for SSDI denial rates) and become statistically significant. However, we caution against drawing conclusions from these estimates, because: 1) there is evidence against the parallel trends assumption, and 2) effects estimated further out in time rely on a select few CZs.

## Discussion

We estimated that the opening of an Amazon FC was associated with a 1.4% reduction in the SSDI applications rate over a 3-year period. This translates to an average of 138 fewer annual applications per commuting zone (95% CI: -405, 138) and 5,528 fewer applications across CZs with an FC per year (95% CI: -16,190, 5,528). Our exploratory results suggest that this reduction was driven by SSDI denials, which is consistent with prior literature that found denial rates linked to fluctuations in local economic opportunity but not approval rates [13, 18]. However, our estimates were not statistically significant, so should be treated as inconclusive.

We note that the confidence intervals upon which the determination of statistical significance were based have been shown to be conservative in simulation studies [34]. Although we do not know of an alternative method by which to estimate confidence intervals, statistical power may be able to be increased by using more recent data. The number of FC openings has accelerated over time (Table 1), but we were unable to estimate the effects of any openings after 2017 due to a lack of SSDI data. It may be of interest to repeat analyses with more recent data, which would result in a larger sample size and more statistical power to detect an effect, should one exist. It is also possible that statistical power was compromised by using the CZ as the unit in the primary analysis, because the number of FC workers would be a very small, possibly insignificant proportion of all workers in a CZ. Given this concern, others [29, 30] have used county as the unit of interest, however, this choice makes the ‘no interference’ identification assumption less tenable. With these trade-offs in mind, we chose to prioritize identification assumptions over statistical power, using CZ as the unit for the primary analysis and county as the unit in a sensitivity analysis.

We hypothesized that the rates of SSDI denials would decrease immediately following an FC opening but that the extent of the decrease would attenuate over time. Although we did see evidence of a decrease in the rate of SSDI denials following an FC opening, that decrease accelerated, rather than attenuated, over time. If the decreases in denial rates and their acceleration are real, one possible reason may be that the opening of FCs result in spillover effects in the local economy that strengthen over time. Although the evidence of spillover effects in terms of employment have been modest and restricted to the warehousing industry [29, 30], to our knowledge, there has been no work estimating the accumulating economic effects using longitudinal estimation approaches that appropriately incorporate time-varying exposure-confounder feedback [46].

We also hypothesized an uptick in SSDI approvals in years following an FC opening due to more workplace injuries and the lack of unionization among Amazon FC workers. However, we did not observe such a signal in the data. This could reflect a true absence of increased injuries, or the truth could be obscured by several factors. First, incomplete information on SSDI decisions in the more recent years could result in measurement error. Second, the FC may represent too small a proportion of the commuting zone’s workforce (a fraction of a percent) to result in a meaningful signal at the commuting zone level. Or third, three years of post-FC data may be too short of a time period to detect effects on injuries. Directly testing the extent to which FC openings affect workplace injuries represents an important area for future work.

Indeed, the small number of post-FC opening years was a limitation. Another limitation was that we were unable to examine possible heterogeneity in effects by relevant subgroups characterized by age, level of education, and race, e.g., or by degree of pre-exisiting economic opportunity across CZs due to a lack of granularity of our outcome data. Others have found important differences in the association between measures of economic opportunity and disability applications [14, 15, 18, 47].

Still another limitation was that a different staggered synthetic control was fit, with different weights, for each outcome (SSDI applications, approvals, and denials). Consequently, we cannot compare results across outcomes. No published method currently exists for extending the partially-pooled staggered adoption SCM to multiple outcomes. It would be of interest to repeat this analysis, fitting one synthetic control for these three outcomes, when such a method is developed.

The synthetic control group approach we used assumes: 1) no interference between units (i.e., CZs); 2) no anticipation, meaning that the intervention has no effect on the outcome prior to implementation; and 3) no unobserved confounding related to treatment timing [34]. If any of these assumptions were violated, then our estimated association may not be a consistent estimate of the causal effect. For example, if the future opening of an FC affected SSDI application rates many months prior to the opening, then this could violate the ‘no anticipation assumption’. In addition, if the 2008 financial crisis (e.g., an example of a macro-level economic shock) affected the timing of FC openings and affected SSDI application rates differently in CZs with FCs versus CZs without FCs, then our estimates could be biased.

To our knowledge this is the first analysis on the potential health effects of Amazon’s expansion. Although our confidence intervals were wide and spanned the null, if our point estimates reflect the truth, our findings are consistent with FC openings improving economic opportunity. Examining other health effects may be informative, including those related to the so-called “deaths of despair” (e.g., drug overdoses, alcohol-related deaths, suicide) [48], as research suggests that “disability applications may itself serve as a sign of rising despair in the face of structural economic change” [17, 18, 22].

## Supporting information

S1 File (PDF)
