# Supplementary material for: Has the opening of Amazon fulfillment centers affected demand for disability insurance?
Source: PLoS One. 2023 Nov 27;18(11):e0294453. doi: 10.1371/journal.pone.0294453 (PMC10681171; doi:10.1371/journal.pone.0294453)
Supplement: S1 File — (PDF) [file pone.0294453.s001.pdf]

Supporting Information for: Has the opening of Amazon fulfillment centers affected demand for disability insurance?

Kara E. Rudolph, Nicholas T. Williams, Floriana Milazzo, Atheendar Venkataramani, and Rourke O'Brien

S1 Covariates

County-level covariates for years 2001-2017 included: median household earnings in the past 12 months (in 2020 inflation-adjusted US dollars), proportion male; proportion white, black, Hispanic/Latino; proportion with the following highest levels of education: less than high school, high school graduate or GED, 4-year college degree or more; population density; proportion in the following age categories: 5-17, 18-24, 25-34, 35-49, 50-64, 65+; proportion of persons 16 and older who were unemployed; proportion of persons 16 and older not in the labor force; proportion of employed civilian population 16 and older in manufacturing jobs, proportion of persons 15 and older never married; proportion of persons 15 and older currently married; proportion of civilian adults ($\geq$ 18 years) who are veterans, all obtained from the US Census Bureau. Years 2010-2017 were obtained from 5-year American Community Survey estimates; for years 2001-2009, we used estimates from the 2000 Census, because 5-year ACS estimates were not available. Lastly, we also used an index of economic mobility as a covariate.Chetty et al. (2014)

Aligned with recommendations for using the synthetic control group approach, we first standardized all covariates.Ben-Michael et al. (2022) Then we reduced the number of covariates to only those that were related to the outcome using lassoTibshirani (1996) (i.e., those retained in the regularized outcome model where the fit is within one standard error of the best fit). This resulted in five covariates being dropped: proportion black; proportion aged 18-24 and 35-49; proportion not in the labor force; and population density.

Table S1: Number of counties with an Amazon Fulfillment Center (FC) in each state that had an FC opening 2006-2017.

State	Number of counties with an FC
Arkansas	11
California	19
Connecticut	2
Florida	13
Georgia	2
Illinois	5
Indiana	28
Kansas	3
Kentucky	24
Maryland	3
Michigan	1
Minnesota	2
Nevada	13
New Jersey	8
Ohio	4
Pennsylvania	38
South Carolina	13
Tennessee	18
Texas	18
Virginia	12
Washington	9
Wisconsin	3

Table S2: Average treatment effect on the treated (ATT) effect estimates and 95% confidence intervals (CIs) by year cohort.

Year of FC opening	ATT over 3 post-opening years	ATT in opening year	ATT 1 year post-opening	ATT 2 years post-opening
	Mean (95% CI)	Mean (95% CI)	Mean (95% CI)	Mean (95% CI)
Applications				
2006	0.008 (-0.012, 0.028)	0.015 (-0.022, 0.041)	0.023 (-0.024, 0.061)	-0.013 (-0.045, 0.025)
2007	-0.031 (-0.160, 0.098)	-0.076 (-0.214, 0.162)	-0.002 (-0.041, 0.061)	-0.015 (-0.063, 0.069)
2008	-0.009 (-0.052, 0.034)	-0.016 (-0.044, 0.019)	-0.014 (-0.054, 0.032)	0.005 (-0.071, 0.071)
2009	—	—	—	—
2010	0.022 (-0.037, 0.081)	-0.001 (-0.023, 0.016)	0.027 (-0.051, 0.083)	0.041 (-0.068, 0.118)
2011	-0.011 (-0.072, 0.050)	-0.017 (-0.077, 0.047)	-0.010 (-0.039, 0.033)	-0.006 (-0.060, 0.072)
2012	-0.019 (-0.084, 0.050)	-0.002 (-0.060, 0.043)	-0.012 (-0.071, 0.047)	-0.043 (-0.134, 0.071)
2013	-0.027 (-0.068, 0.014)	-0.023 (-0.053, 0.010)	-0.012 (-0.044, 0.021)	-0.046 (-0.105, 0.024)
2014	-0.005 (-0.044, 0.034)	0.008 (-0.068, 0.063)	0.006 (-0.039, 0.039)	-0.028 (-0.075, 0.021)
2015	-0.008 (-0.075, 0.059)	0.000 (-0.036, 0.042)	-0.009 (-0.058, 0.059)	-0.014 (-0.084, 0.093)
2016	-0.026 (-0.108, 0.056)	-0.026 (-0.093, 0.055)	-0.025 (-0.103, 0.071)	—
2017	-0.024 (-0.106, 0.058)	-0.024 (-0.099, 0.063)	—	—
Denials				
2006	0.011 (-0.014, 0.036)	0.001 (-0.061, 0.070)	0.031 (-0.017, 0.075)	0.001 (-0.034, 0.034)
2007	-0.062 (-0.299 0.175)	-0.096 (-0.278, 0.199)	-0.043 (-0.160, 0.156)	-0.046 (-0.175, 0.171)
2008	-0.044 (-0.177, 0.089)	-0.043 (-0.124, 0.079)	-0.058 (-0.166, 0.094)	-0.031 (-0.154, 0.118)
2009	—	—	—	—

Figure S1: Estimated difference between log-transformed SSDI (a) application rates, (b) denial rates, and (c) approval rates comparing commuting zones with an FC opening 2011 and later to their synthetic control commuting zones without an FC by year. The synthetic control was a weighted combination of commuting zones without an FC such that the weights minimized the difference in outcome and covariates between FC and non-FC commuting zones in the years preceding an FC opening. The average line to the right of the vertical line represents the effect of an FC opening for the 3 years after the opening, with 95% confidence intervals.

(a) SSDI Application rates

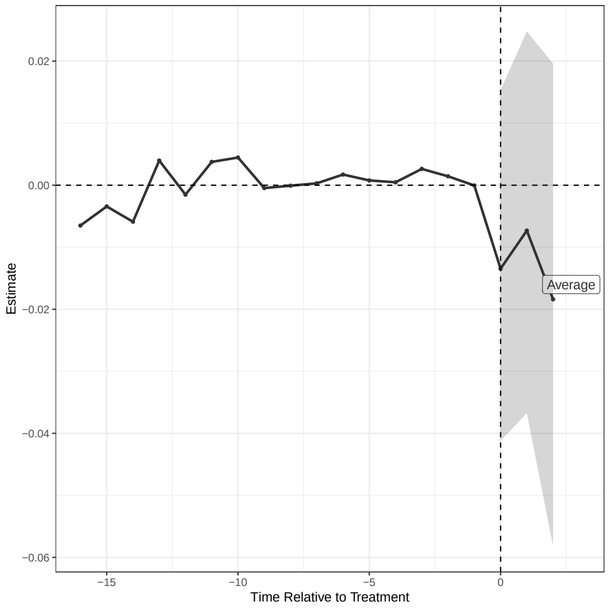

(b) SSDI Denial rates

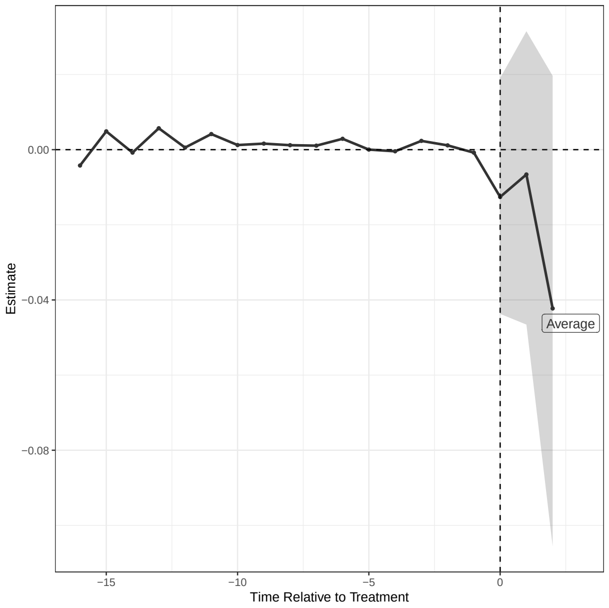

(c) SSDI Allowance rates

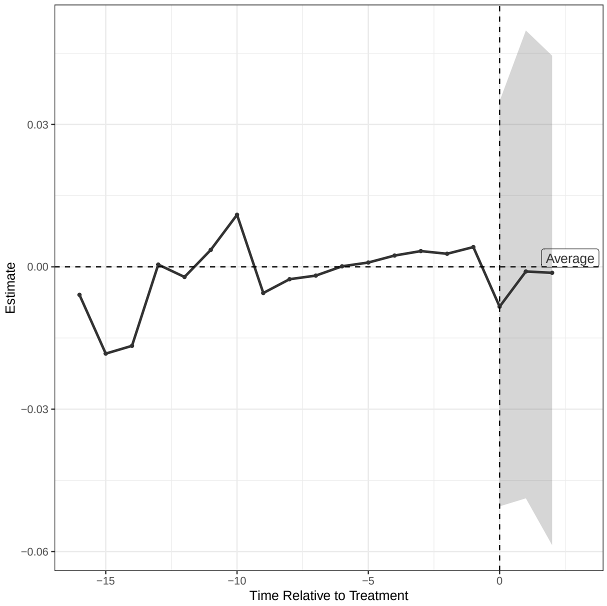

Figure S2: Estimated difference between log-transformed SSDI application rates comparing commuting zones with an FC opening to their synthetic control commuting zones without an FC by year setting the pooling parameter that determines the relative weights given to the separate SCM and pooled SCMto be a) 0.1 and b) 0.9. The pooling parameter in the primary analysis is 0.27. The synthetic control was a weighted combination of commuting zones without an FC such that the weights minimized the difference in outcome and covariates between FC and non-FC commuting zones in the years preceding an FC opening. The average line to the right of the vertical line represents the effect of an FC opening for the 3 years after the opening, with 95% confidence intervals.

(a) Pooling parameter=0.1 (b) Pooling parameter=0.9

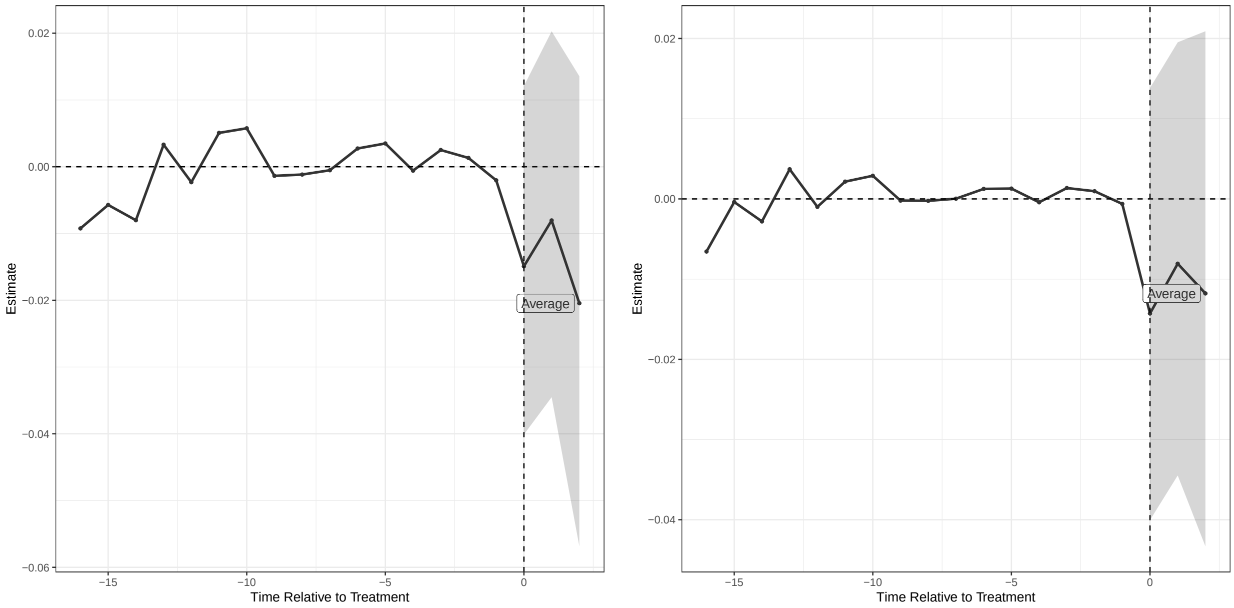

Figure S3: Placebo test: Estimated difference between log-transformed SSDI application rates comparing commuting zones with an FC opening but with a fake opening date (5 years prior to the true opening date) to their synthetic control commuting zones without an FC by year. The average line to the right of the vertical line represents the effect of an FC opening for the 3 years after the opening, with 95% confidence intervals.

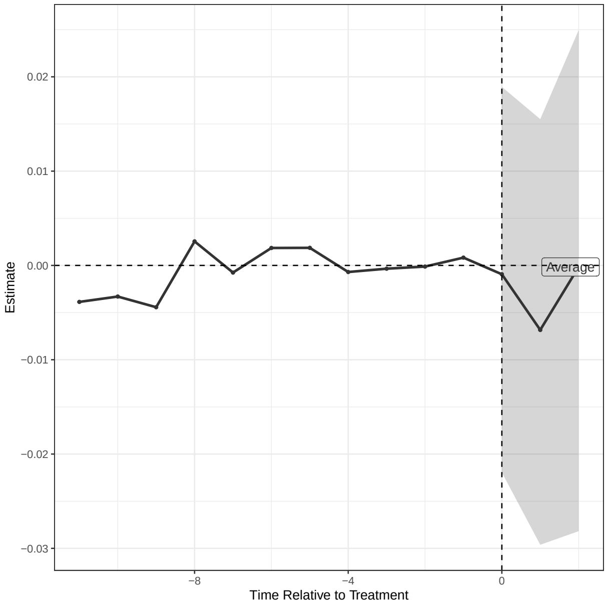

Figure S4: County analysis: Estimated difference between log-transformed SSDI (a) application rates, (b) denial rates, and (c) allowance rates comparing counties with an FC opening to their synthetic control counties without an FC by year. The synthetic control was a weighted combination of counties without an FC such that the weights minimized the difference in outcome and covariates between FC and non-FC counties in the years preceding an FC opening. The average line to the right of the vertical line represents the effect of an FC opening for the 3 years after the opening, with 95% confidence intervals.

(a) SSDI Application rates

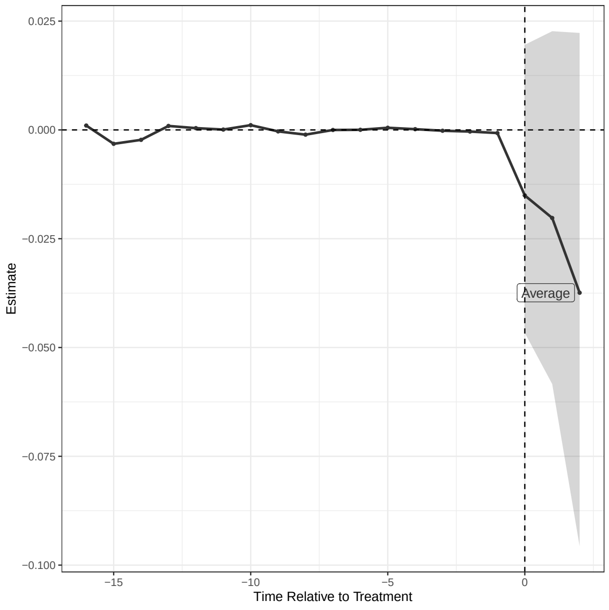

(b) SSDI Denial rates

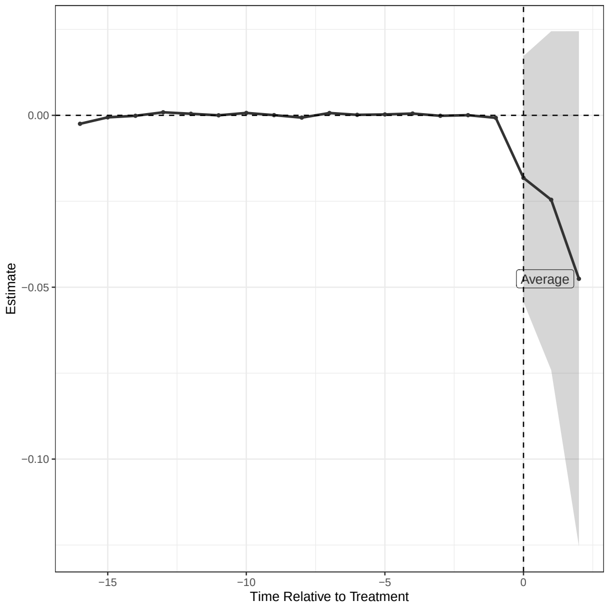

(c) SSDI Allowance rates

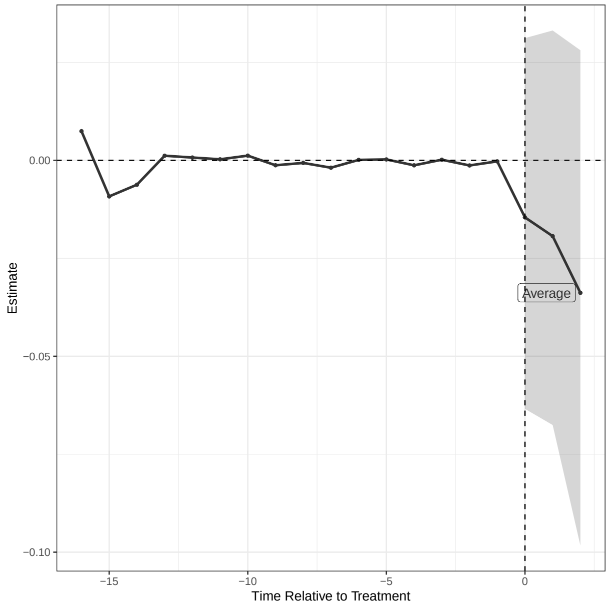

Figure S5: Estimated difference between log-transformed SSDI current beneficiary rates comparing commuting zones with an FC opening to their synthetic control commuting zones without an FC by year. The synthetic control was a weighted combination of commuting zones without an FC such that the weights minimized the difference in outcome and covariates between FC and non-FC commuting zones in the years preceding an FC opening. The average line to the right of the vertical line represents the effect of an FC opening for the 7 years after the opening, with 95% confidence intervals.

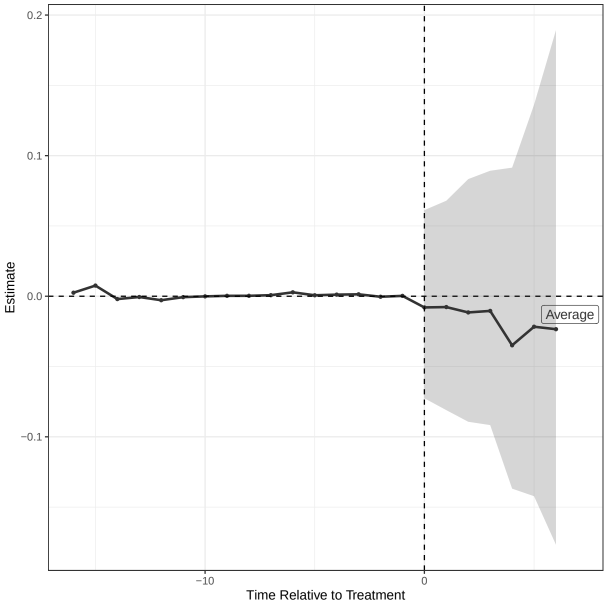

Figure S6: Estimated difference between log-transformed SSDI (a) application rates, (b) denial rates, and (c) allowance rates comparing CZs with an FC opening to their synthetic control CZs that have not yet had an FC opening but will in the future. The synthetic control was a weighted combination of CZs that had not yet opened an FC but would open one in the future such that the weights minimized the difference in outcome and covariates between FC and non-FC-yet CZs in the years preceding an FC opening. The average line to the right of the vertical line represents the effect of an FC opening for the 3 years after the opening, with 95% confidence intervals.

(a) SSDI Application rates

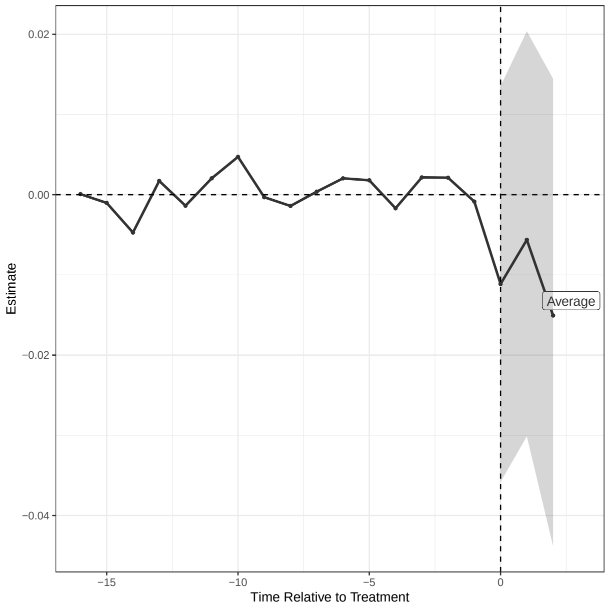

(b) SSDI Denial rates

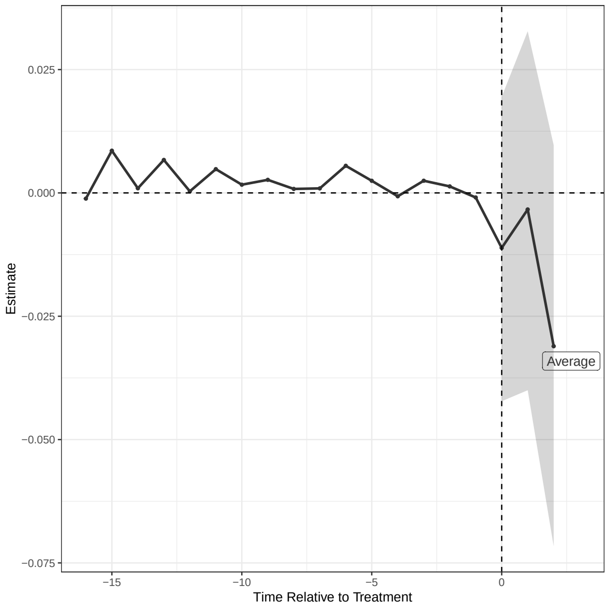

(c) SSDI Allowance rates

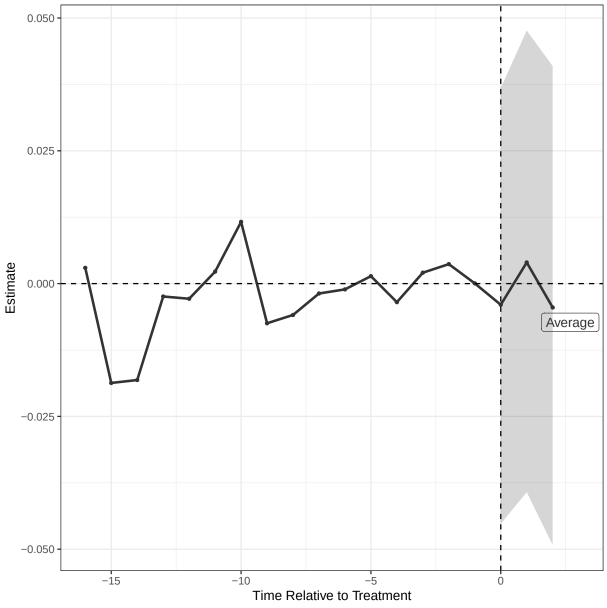

Figure S7: Estimated time-cohort difference-in-differences estimates of the ATT for the effect of FC openings in a CZ on log-transformed SSDI (a) application rates, (b) denial rates, and (c) allowance rates. Estimates and 95% confidence intervals for the 3 years after the FC opening.

(a) SSDI Application rates

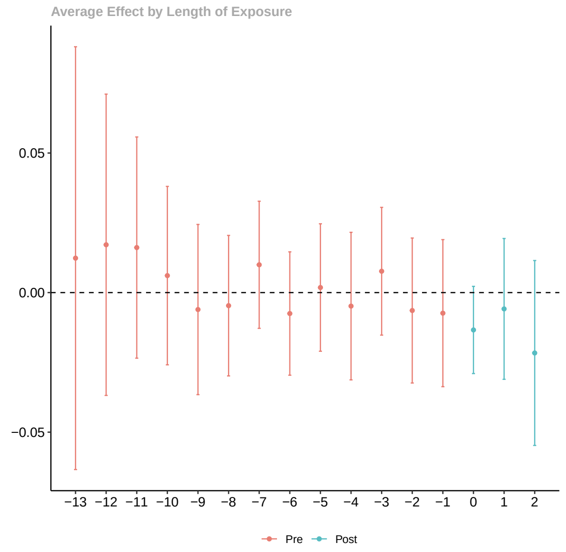

(b) SSDI Denial rates

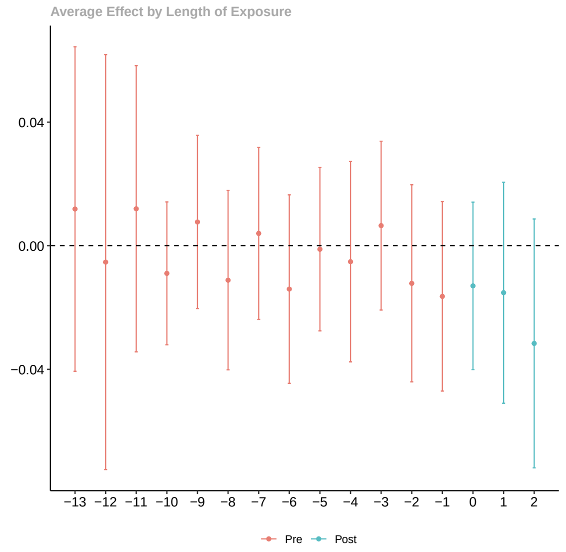

(c) SSDI Allowance rates

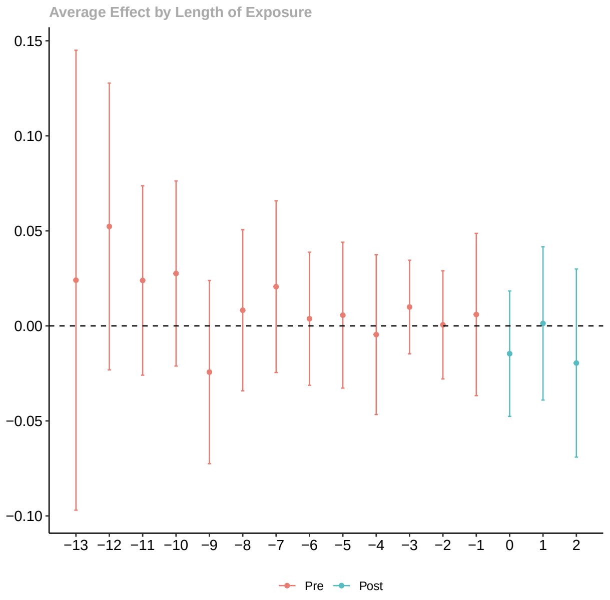

Figure S8: Estimated time-cohort difference-in-differences estimates of the ATT for the effect of FC openings in a CZ on log-transformed SSDI (a) application rates, (b) denial rates, and (c) allowance rates. Estimates and 95% confidence intervals for all years after the FC opening.

(a) SSDI Application rates

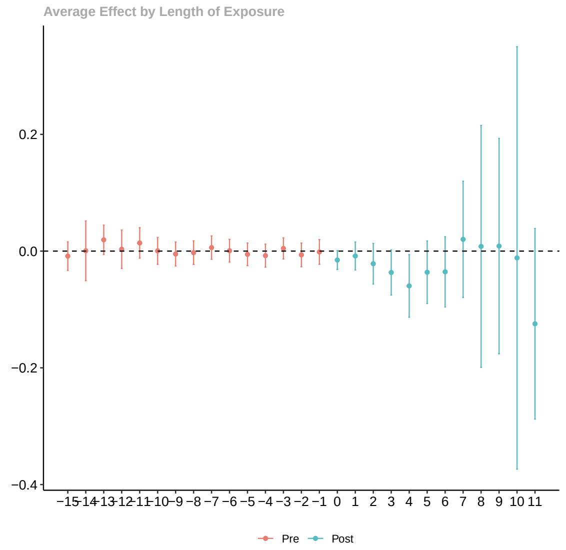

(b) SSDI Denial rates

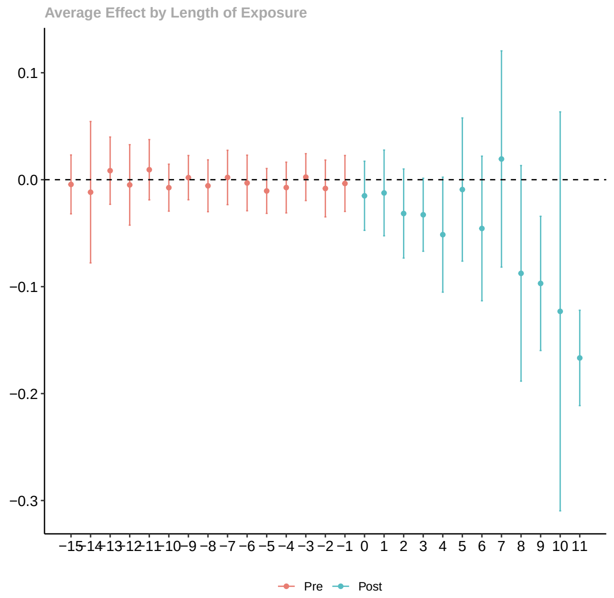

(c) SSDI Allowance rates

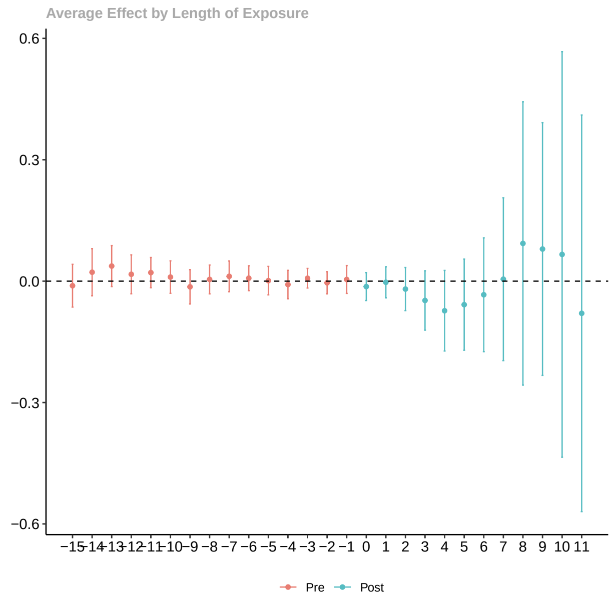

References

Ben-Michael, E., Feller, A., Rothstein, J., et al. (2022). Synthetic controls with staggered adoption. *Journal of the Royal Statistical Society Series B*, 84(2):351–381.

Chetty, R., Hendren, N., Kline, P., and Saez, E. (2014). Where is the land of opportunity? the geography of intergenerational mobility in the united states. *The Quarterly Journal of Economics*, 129(4):1553–1623.

Tibshirani, R. (1996). Regression shrinkage and selection via the lasso. *Journal of the Royal Statistical Society: Series B (Methodological)*, 58(1):267–288.
